# Smoking Doubles the Mortality Risk in COVID-19: A Meta-Analysis of Recent Reports and Potential Mechanisms

**DOI:** 10.7759/cureus.10837

**Published:** 2020-10-07

**Authors:** Husam M Salah, Tanya Sharma, Jawahar Mehta

**Affiliations:** 1 Internal Medicine, University of Arkansas for Medical Sciences, Little Rock, USA

**Keywords:** smoking, covid-19, mortality, sars-cov-2

## Abstract

Introduction

Studies have reported conflicting results regarding the effect of smoking on outcome in coronavirus disease 2019 (COVID-19) patients, but the results have been conflicting. In this meta-analysis, we systematically examined the association between smoking and mortality in COVID-19.

Methods

PubMed database was searched to look for relevant articles. Inclusion criteria were as follows: (1) cohort studies or case series studies; (2) study population included individuals with a confirmed COVID-19 infection; (3) the status of smoking was reported, regardless if it was current or in the past; and (4) mortality among smokers was reported in the study or could be calculated and compared to non-smokers. Mortality rates were pooled using a random effects model. Risk ratio (RR) and its 95% confidence interval (CI) were also calculated using the same model. Another meta-analysis was then performed to assess the difference in mortality between current and former smokers.

Results

Ten studies with a total of 11,189 patients were included. Mortality among smokers was 29.4% compared to 17.0% among non-smokers. RR was 2.07 (95% CI: 1.59, 2.69). Based on analysis of four studies (532 patients), there was no difference in mortality risk between current and former smokers (RR: 1.03; 95% CI: 0.75, 1.40).

Conclusions

Smoking, current or past, is associated with higher mortality in COVID-19 patients. Mortality among current smokers was about 50% greater than former smokers, but the difference was not statistically significant.

## Introduction

The coronavirus disease 2019 (COVID-19), caused by the severe acute respiratory coronavirus 2 (SARS-CoV-2), is a current active pandemic and major healthcare concern. The mortality rate among all patients with COVID-19 infection might reach up to 15.2%, and as high as 53.4% among patients in the intensive care unit [1,2]. Our understanding of factors associated with higher mortality is still evolving. Recent reports show that mortality is higher among patients with hypertension, chronic obstructive pulmonary disease (COPD), hypercholesterolemia, diabetes mellitus (DM), and cardiovascular disease, as well as in older men [2,3]. There is intense interest in studying the impact of modifiable host factors, such as smoking, on the severity of COVID-19 infection for early identification of individuals at higher risk of mortality and targeted prevention of adverse outcomes.

Smoking has been reported to be a risk factor for developing acute respiratory distress syndrome (ARDS) and is associated with higher intensive care costs in a dose-dependent fashion [4,5]. Theoretically, smoking could impact outcomes of COVID-19 patients directly by enhancing inflammation and impairing endothelial function in the cardiopulmonary systems. However, studies related to the effect of smoking on the severity of COVID-19 infection have reported conflicting results. While some studies reported no significant association between smoking and the severity of the disease in patients with COVID-19 [6], others reported a more severe form of the disease among smokers [7-9].

We examined the effect of smoking on mortality in a large cohort of patients with COVID-19 infection.

## Materials and methods

Search strategy

A systematic review was conducted in the PubMed database to identify articles that examined the association between smoking and COVID-19 using the following keywords: (“smoking” and “COVID-19”), (“smoker” and “COVID-19”), and (“smokers” and “COVID-19”) from inception until July 26, 2020. No language restriction was applied.

Study selection

For the meta-analysis, only studies that compared mortality between smokers with COVID-19 infection and non-smokers with COVID-19 infection were included. Inclusion criteria were as follows: (1) cohort studies; (2) study population included individuals with a confirmed COVID-19 infection; (3) the status of smoking was reported, regardless if it was current or in the past; and (4) mortality among smokers was reported in the study or could be calculated and compared to non-smokers. All other studies were excluded.

Data extraction

For each study, the data screening and abstraction were performed by two independent authors (H.S. and T.S.). All discrepancies were resolved by consensus.

Data analysis

The pooled sample was divided into two groups: (1) patients with COVID-19 infection who were current or former smokers and (2) patients with COVID-19 infection who never smoked. Mortality rates from the included studies were pooled using a random-effect model, which assumes that the included studies in our meta-analysis represent a random sample of effect sizes. Risk ratio (RR) and its 95% confidence interval (CI) were calculated using the same model. Cochran's Q and *I*^2^ index were used for heterogeneity estimation. An *I*^2^ index of <25% was considered low heterogeneity, whereas an *I*^2^ index of >80% was considered high heterogeneity. An *I*^2^ index between 25% and 80% was considered moderate heterogeneity. We used a funnel plot to test for potential publication bias. Sensitivity analysis was performed by excluding the two studies with the largest weight. All statistical analysis was performed using the Review Manager 5.4 Software.

## Results

**Figure 1:**
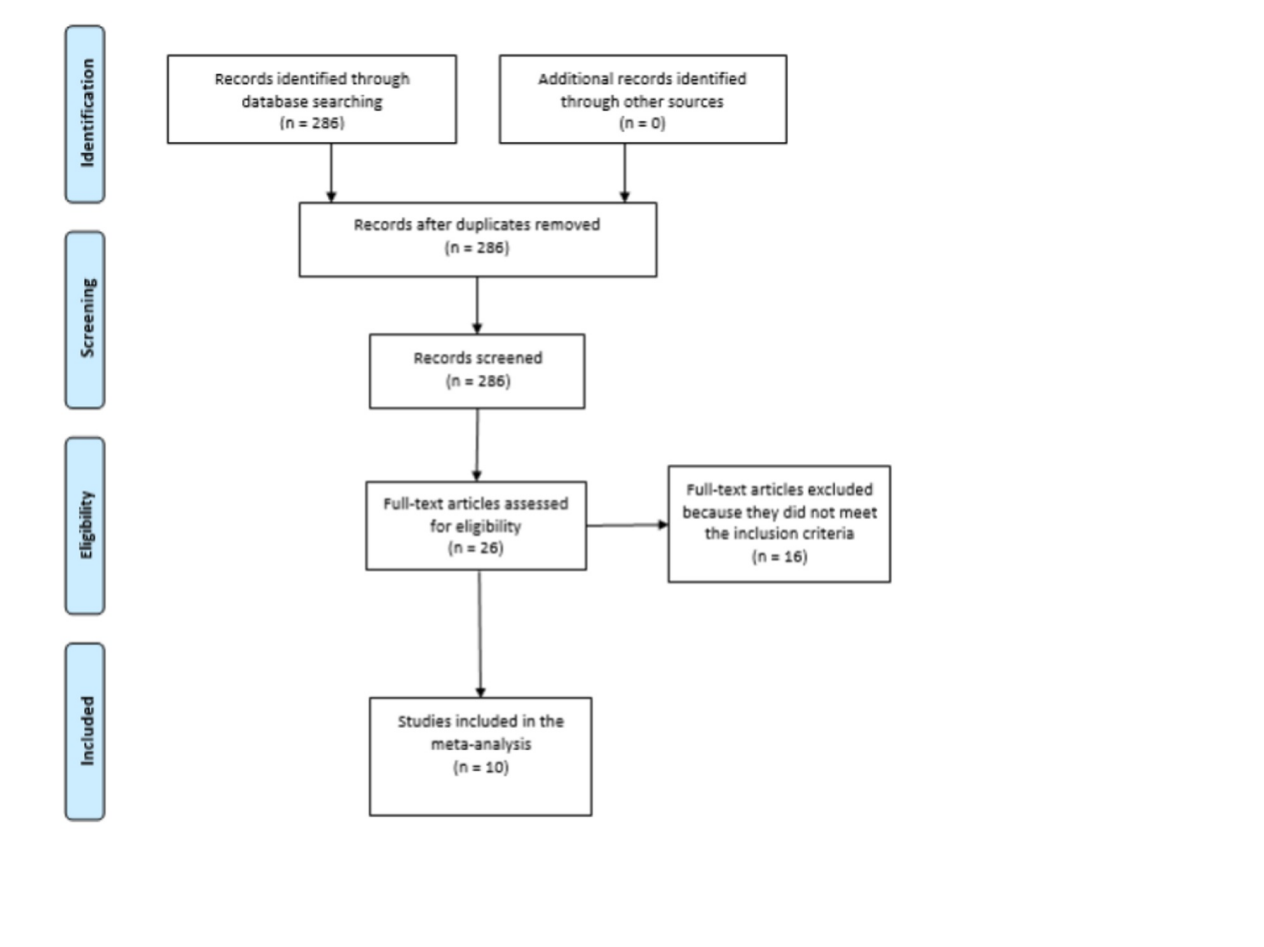
PRISMA flowchart for the study inclusion and exclusion. PRISMA, Preferred Reporting Items for Systematic Reviews and Meta-Analyses

**Figure 2:**
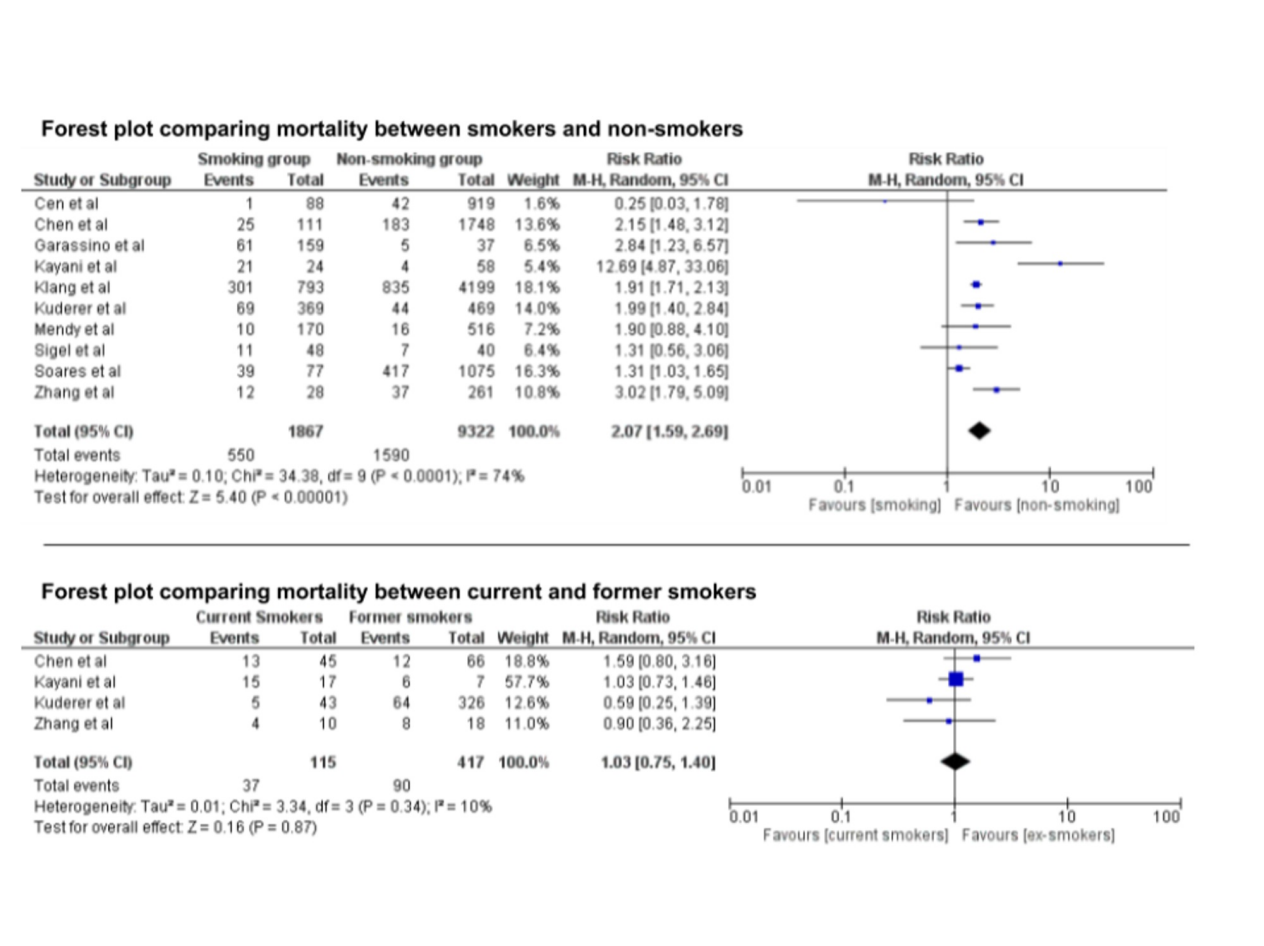
Forest plots comparing mortality between smokers and non-smokers among patients with COVID-19 (upper) and mortality between current and former smokers (lower). COVID-19, coronavirus disease 2019

**Figure 3:**
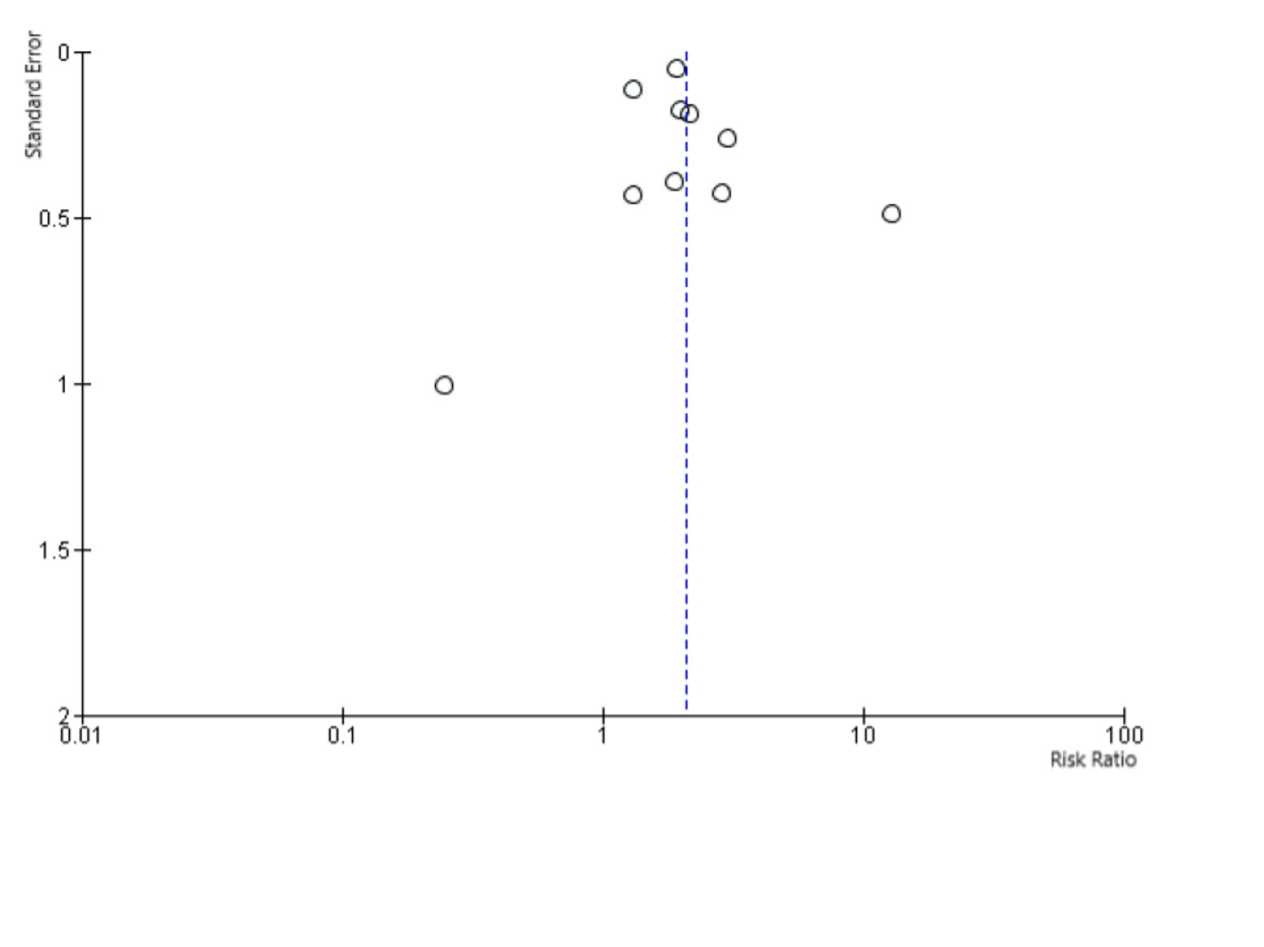
Funnel plot to assess for potential publication bias.

We initially identified 286 studies. After careful assessment by applying the inclusion criteria, only 10 studies with a total of 11,189 patients were included (Figure 1). Out of those, 1,867 patients were smokers, current or past. Mortality among smokers was 29.4% compared to 17.0% among non-smokers. RR was 2.07 (95% CI: 1.59, 2.69). *I*^2^ index was 74%, suggesting moderate heterogeneity. Sensitivity analysis yielded consistent results. In the meta-analysis that compared mortality between current and former smokers, only four studies were included with a total of 532 patients. Mortality among current smokers was 32.1%, whereas mortality among former smokers was 21.5%. RR was 1.03 (95% CI: 0.75, 1.40), suggesting a non-statistically significant increase. *I*^2^ index was 10%, suggesting low heterogeneity (Figures 2, 3).

## Discussion

Although there are several reports of higher mortality in smokers who have concurrent COVID-19 infection, we conducted a systematic analysis using strict inclusion criteria of the effect of current and past smoking on mortality in this high-risk population.

In this large meta-analysis, 16.7% of included patients were either current or former smokers. Smoking, current or past, was associated with a nearly two-fold higher mortality rate among COVID-19 patients.

**Figure 4:**
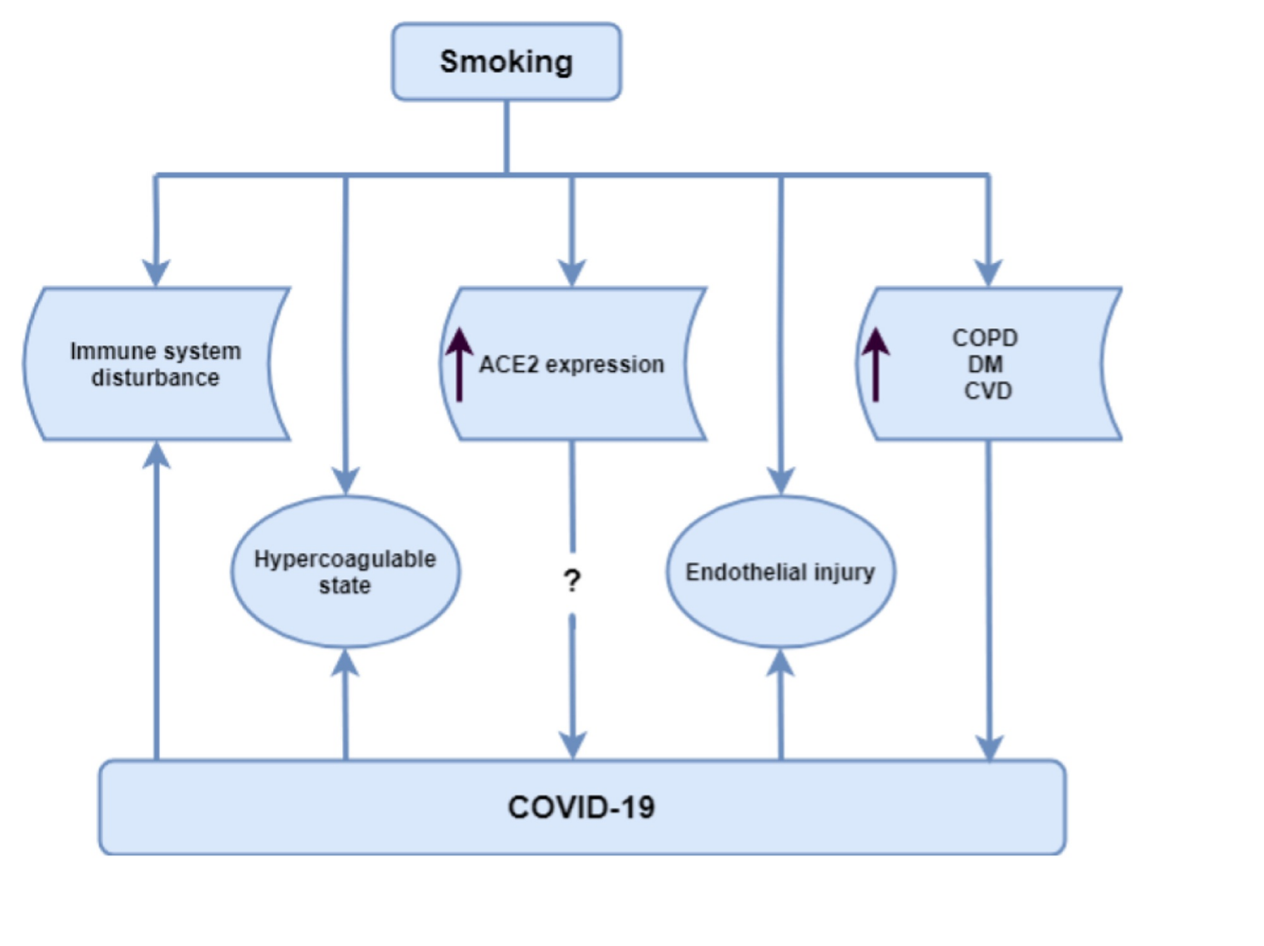
Mechanisms that might explain the higher mortality rate among smokers with COVID-19 infection compared to non-smokers. ACE2, angiotensin-converting enzyme 2; COPD, chronic obstructive pulmonary disease; COVID-19, coronavirus disease 2019; CVD, cardiovascular disease; DM, diabetes mellitus

This association of smoking with higher mortality in COVID-19 patients can be explained by several mechanisms. These potential mechanisms are graphically shown in Figure 4.

Smokers are more prone to more severe infections due to poor mucociliary clearance and an exaggerated cellular response marked by oxidative stress, increased permeability, mucus overproduction, and release of pro-inflammatory cytokines [10,11]. Smokers are therefore more likely to develop ARDS and do worse with respiratory diseases owing to a lowered pulmonary reserve and altered physiology.

SARS-CoV-2 utilizes angiotensin-converting enzyme 2 (ACE2) receptors to gain entry into the respiratory epithelial cells [12]. A recent study has shown that smoking increases the expression of ACE2 in the secretory cells of the respiratory tract [13]. Although the association between ACE2 expression and the disease severity in COVID-19 patients is still not well understood, it is possible that high ACE2 expression in smokers might play a role in the adverse outcome observed among this population by either direct epithelial cell damage or through downstream events of the inflammatory cascade.

Smoking can alter the innate and adaptive immune systems and can result in exacerbation of pathological immune responses and attenuation of normal physiological functions of the immune system [14]. Additionally, the redox imbalance associated with smoking can activate nuclear-factor-kB and activator protein-1, which lead to gene expression of pro-inflammatory cytokines, such as interleukin-6, interleukin-8, and tumor necrosis factor-alpha. Evolving evidence supports the concept that uncontrolled overproduction of similar inflammatory mediators, i.e., “cytokine storm”, plays a crucial role in the occurrence of ARDS in patients with COVID-19 [15,16]. The occurrence of this cytokine storm is associated with worse outcomes in COVID-19 infection and is another mechanism by which smoking can contribute to higher mortality among these patients [17].

Additionally, smoking can cause pulmonary endothelial damage and, thus, an abnormal barrier function [18]. This can subsequently increase alveolar permeability and the risk of pulmonary edema. Smoking further contributes to vascular dysfunction by promoting a hypercoagulable state and is an independent risk factor for thromboembolism [19]. COVID-19 has been noted to be associated with a hypercoagulable state, which is associated with worse outcomes [20].

In addition to the direct pathological cascade associated with smoking, the presence of pre-existing comorbidities seems to contribute to worse outcomes. Smoking is an independent risk factor for developing COPD, DM, and cardiovascular disease [21-23]. In multiple studies, the presence of these comorbidities led to poor outcomes and more severe disease in COVID-19 [2,3,8,19].

While our analysis shows worse in-hospital and immediate mortality among smokers with COVID-19 infection as compared to non-smokers, we would anticipate higher long-term morbidity among COVID-19 survivors who currently smoke. In a study investigating six-month health utility outcomes among survivors of ARDS, smoking is associated with worse quality of life among this population [24]. This would be of interest as we attempt to understand more about the sequelae of COVID-19 infection and the interplay of acute infection with pre-existing health factors.

There are several limitations of the results of this meta-analysis. First, all meta-analyses have the inherent limitations of including studies with different populations, although we tried to include studies with similar patient populations as indicated by *I*^2^ index of 74%, suggesting moderate heterogeneity. Second, there may well be an interaction between smoking and the prevalence of other co-morbidities, such as DM and hypertension, which was not studied. Lastly, we could not study the quantity and duration of smoking with increased mortality in COVID-19 patients in our analysis.

## Conclusions

Despite the inherent limitation of this meta-analysis, we show that smoking is associated with a significantly higher risk of mortality in patients with COVID-19. Although the difference in mortality between current and past smokers was not statistically significant, it appeared to be about 50% greater in current smokers.
